# The effect of magnesium on early osseointegration in osteoporotic bone: a histological and gene expression investigation

**DOI:** 10.1007/s00198-017-4004-5

**Published:** 2017-03-27

**Authors:** S. Galli, M. Stocchero, M. Andersson, J. Karlsson, W. He, T. Lilin, A. Wennerberg, R. Jimbo

**Affiliations:** 1 Department of Prosthodontics, Faculty of Odontology, Malmö University, 205 06 Malmö, Sweden; 2 Department of Oral and Maxillofacial Surgery and Oral Medicine, Faculty of Odontology, Malmö University, Malmö, Sweden; 3 Department of Chemistry and Chemical Engineering, Applied Chemistry, Chalmers University of Technology, 412 96 Gothenburg, Sweden; 4 Center for Biomedical Research, ECole Nationale Vétérinaire d’Alfort, 94700 Maisons Alfort, France

**Keywords:** Implant surface, In vivo, Magnesium, Mesoporous titania, Osseointegration, Osteoporosis

## Abstract

**Summary:**

Magnesium has a key role in osteoporosis and could enhance implant osseointegration in osteoporotic patients. Titanium implants impregnated with Mg ions were installed in the tibia of ovariectomized rats. The release of Mg induced a significant increase of bone formation and the expression of anabolic markers in the peri-implant bone.

**Introduction:**

The success of endosseous implants is highly predictable in patients possessing normal bone status, but it may be impaired in patients with osteoporosis. Thus, the application of strategies that adjuvate implant healing in compromized sites is of great interest. Magnesium has a key role in osteoporosis prevention and it is an interesting candidate for this purpose. In this study, the cellular and molecular effects of magnesium release from implants were investigated at the early healing stages of implant integration.

**Methods:**

Osteoporosis was induced in 24 female rats by means of ovariectomy and low-calcium diet. Titanium mini-screws were coated with mesoporous titania films and were loaded with magnesium (test group) or left as native (control group). The implants were inserted in the tibia and femur of the rats. One, 2 and 7 days after implantation, the implants were retrieved and histologically examined. In addition, expression of genes was evaluated in the peri-implant bone tissue at day 7 by means of quantitative polymerase chain reactions with pathway-oriented arrays.

**Results:**

The histological evaluation revealed that new bone formation started already during the first week of healing for both groups. However, around the test implants, new bone was significantly more abundant and spread along a larger surface of the implants. In addition, the release of magnesium induced a significantly higher expression of BMP6.

**Conclusions:**

These results provide evidence that the release of magnesium promoted rapid bone formation and the activation of osteogenic signals in the vicinity of implants placed in osteoporotic bone.

**Electronic supplementary material:**

The online version of this article (doi:10.1007/s00198-017-4004-5) contains supplementary material, which is available to authorized users.

## Introduction

Osteoporosis is a skeletal disease characterized by decrease in bone mass and density and abnormalities in bone microarchitecture, which results in increased susceptibility to fractures [1]. Osteoporosis is a relevant public health issue, which affects up to 50% of women and 20% of men in the white population over the age of 50 [1]. The prevalence increases with age and is gender-related and on the basis of radiographic evidence almost all observed women by the age of 75 show a moderate to severe osteoporosis [2].

Bone mineral density (also referred to as “bone mass”) is the result of genetic asset, but it is also strongly influenced by environmental factors, such as availability of calcium and vitamin D, mechanical loading and age [3]. A decrease in bone mass is observed when an uncoupling of bone formation and bone resorption occurs during the physiological process of skeletal remodelling, with a prevalence of the resorption phase over the formation phase [1]. The first recognized etiological factor for this type of bone loss is oestrogen deficiency. Oestrogens have a regulatory role on bone remodelling, and they control especially the differentiation and activation of osteoclasts through several molecular pathways, keeping bone resorption within physiological range [4]. In women after menopause, due to the decrease in oestrogen levels, the oestrogen-mediated regulation is impaired and the osteoclast activity raises [4]. However, other oestrogen-independent mechanisms, mostly of which are age-related, can induce bone loss as well [5].

Osteoporosis may represent a risk for the osseointegration of endosseous implants [6–8] as bone may be too rarefied to offer good implant primary stability, one of the pre-requisites for osseointegration [9].

Although data from available literature suggests that osteoporosis is not an absolute contraindication to oral and orthopaedic implant placement [10, 11], there is certain evidence that this disorder may increase the rate of implant failure.

It has been shown that postmenopausal women had statistically significantly higher implant failure in the maxilla compared to premenopausal women and men older than 50 (13.6% of failure vs. 6.3 and 7.6%) [12]. However, if the osteoporotic women had received oestrogen supplementation, their implant failure rate was lower than in postmenopausal women without hormone replacement treatment (13.6 vs. 8.1%) [12]. No difference was observed for implant placed in the mandible [12].

Furthermore, evidence exists that osteoporosis could represent a threat to osseointegration when associated with other risk factors, such as smoking, diabetes mellitus or bruxism, which together may increase considerably the risk of implant failure [6].

Therefore, the development of protocols and materials targeted to improve performances in patients with osteoporosis is still of primary interest.

One strategy to improve the long-term fixation of implants in osteoporotic sites has been to functionalize the implant surfaces, to stimulate peri-implant osteogenesis [13].

A promising approach in this scenario has been the inclusion of bone resorption inhibitors of the bisphosphonate class (raloxifene and alendronate) loaded into mesoporous coatings deposited onto titanium implants. The coatings provided a sustained release of the drugs directly at the healing site with the effect of enhancing the bone anchorage of the implants in vivo [14, 15].

However, a potential risk of bisphosphonates is to induce osteonecrosis, a condition documented especially in the jaws [16]. Although this adverse effect has been so far associated only with high doses of the drugs in systemic applications, no clinical evidence exists that indicates local application as a risk-free solution; therefore, an alternative doping material would be of great interest [17].

One of the possible candidates for doping implant surfaces is magnesium (Mg), which has been reported to significantly increase the bone-implant fixation in vivo and in clinical situations [18–20]. In previous studies, we have documented the osteoconductive and osteogenic potential of magnesium when released from mesoporous titania surfaces and confirmed the associated increase of bone formation and biomechanical anchorage [21–23].

Of particular interest is whether magnesium availability has a key role in osteoporosis. Magnesium is strongly involved in bone metabolism, both as a mitogen factor for osteoblasts, which proliferate in the presence of magnesium, and as protective factor from excessive bone resorption [24]. Magnesium deficiency stimulates osteoclasts activation, due to free radicals production [25], and it has been correlated to impaired bone growth, skeletal fragility and osteoporosis in rats and humans [26]. Potential mechanisms of lower bone formation when magnesium is reduced are the reduced synthesis of parathyroid hormone and of vitamin D [27]. Moreover, the mineral metabolism and the calcification in bone are influenced by Mg ions [28] and abnormal mineralization may occur when serum levels of magnesium is low [27].

Recently, magnesium supplementation has been shown as essential as calcium and vitamin D for bone health, acting as a protective factor from osteoporosis and osteoporosis-related fractures, [29].

Therefore, magnesium may be a good alternative as adjuvant on implants surfaces, especially in patients with compromized bone conditions. Thus, the objective of the current study was to investigate the effects of magnesium during the initial phases of implant healing and in particular, to study the biological pathways that are involved in the bone healing in compromized conditions using both histological and gene expression evaluation techniques.

## Material and methods

### Implants

Mini-screws of titanium grade IV (Neodent, Curitiba, Brazil), with 1.5 mm diameter and 2.5 mm length, were used for this study. The implants had originally a turned surface, as indicated by the manufacturer.

The screws were coated with a TiO_2_ layer, through the evaporation-induced-self-assembly (EISA) method, as previously described [22, 30].

In brief, a Pluronic^®^ P123 template was used as precursor and was diluted in absolute ethanol. A titania precursor mixture containing titanium(IV)ethoxide (Aldrich) and hydrochloric acid (HCl, 37%, Aldrich) was added and mixed. The mixture was deposited on the mini-screws by spin coating for 1 min at 7500 rpm. Thereafter, the surfaces were dried overnight to allow complete self-assembly of the TiO_2_ structure. The samples were then heated from room temperature to 350 °C (temp increase 1 °C/min). Once reached 350 °C, the samples were kept at this temperature for 4 h.

This method has demonstrated to produce a mesoporous titania layer with a 3D cubic structure, with pores of approximately 6 nm in diameter [31].

The coated screws were immersed in magnesium chloride (MgCl_2_) solution at 10 mg/ml concentration, serving as the source of magnesium ions, which were deposited into the mesoporous matrix by physical deposition. The Mg ion deposition was performed on 50 screws, while the other 50 were left as native mesoporous titania, without Mg addition, and served as controls.

After preparation, the surfaces were characterized using scanning electron microscopy (SEM) with a Leo Ultra55 FEG Instrument (Zeiss, Oberkochen, Germany) at an accelerating voltage of 5 kV. Chemical analysis of the surface was performed with energy dispersive spectroscopy (EDX) at a voltage of 12 kV.

In addition, the quantity of Mg ions absorbed in the coating was analysed by inductively coupled plasma-sector field mass spectrometry (ICP-SFMS). Three implants loaded with Mg ions and three control implants were used for this analysis. The implants were immersed in 70% nitric acid (HNO_3_) for 24 h. The immersing media was then analysed with ICP-SFMS.

### Animal experiment

The current animal study in rats was approved by the French ministry for research after ethical review in accordance with the European directive 2010–63 and conducted at the Centre De Recherce Biomédicale, Ecole National Vétérinaire d’Alfort, Maisons Alfort, France.

Twenty-four Sprague Dawley female rats, 6 months old, were included in this study. Osteoporosis-like conditions were induced in the rats through ovariectomy and a low-calcium diet. In brief, the rats were anesthetized through inhalation of isoflurane 1% dissolved in O_2_. The skin of the abdomen was shaved and then cut along the *linea alba*. The abdominal muscles were dissected to access the peritoneal cavity. The ovaries were located and exposed by gentle retraction. Thereafter, the uterine tube and the ovaries were identified, and finally a Vicryl 4.0 suture was sutured around the distal part of the tubes. The ovaries were thereafter cut and removed.

The animals were provided with antibiotics and analgesics after the surgery. They were kept two or three per cage and were allowed to move freely.

Post-operatively, the rats were fed a low-calcium diet, which contained 0.1–0.2% of calcium, in order to induce an osteoporotic situation. This diet was continued throughout the experimental period.

Three months after ovariectomy, the rats were anesthetized with the same procedure. An incision was made in the skin over the medial face of the tibia and femur. A full-thickness periosteal flap was elevated and the medial tibia plate, as well as the femoral distal medial epicondyle, was exposed. Osteotomies were created with a sequence of 1-mm- and 2-mm-diameter burs, under constant irrigation of sterile saline solution. The mini-screws with or without magnesium doping (test and control groups) were randomly allocated to both sides of the hind limbs. Each rat received one screw in each tibia and one screw in each femur, for a total of four screws per animal, two tests and two controls.

After 1, 2, and 7 days of healing, the rats were euthanized with a pentobarbital overdose.

After 1 and 2 days of healing, the implants and the surrounding bone were explanted from the tibiae and femurs and fixed in 70% ethanol for further histological processing.

At the 7-day sacrifice, the implants in the tibia were explanted and fixed with 70% ethanol for further histological examinations, while the implants in the femurs were retrieved together with a small amount of surrounding tissue and were placed in *RNAlater®* buffer (LifeTechnologies, Thermo Fisher Scientific, Waltham, USA) and stored at −80 °C until further processing.

### Non-decalcified histology

The explants preserved in 70% ethanol were de-hydrated in ascending ethanol concentrations for 4 weeks. Afterwards, infiltration in methylmetacrylate resin (Technovit 7200, VLC; Hereaeus Kulzer, Wehrheim, Germany) was performed for 5 weeks. The samples were then embedded in the same resin and polymerized under UV-light for 3.5 h.

The samples were then cut along the longitudinal axis of the implants with Exakt saw (Exakt Apparatebau, Nordertedt, Germany) and were grinded down to a thickness of approximately 40 μm.

The sections were stained with toluidine blue-pyronin g (Histolab, Göteborg, Sweden). They were then observed at the optical light microscope (Eclipse ME600; Nikon, Tokyo, Japan).

**Fig. 1:**
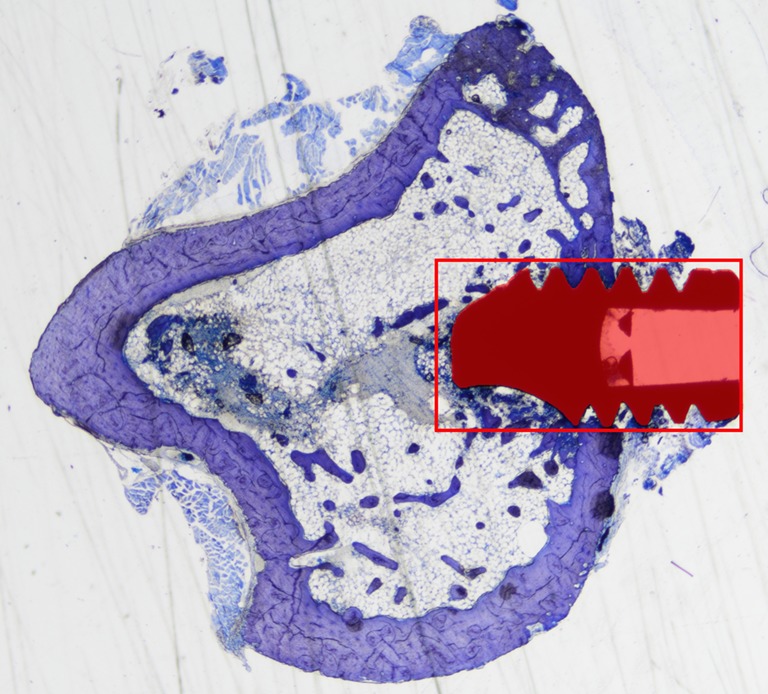
Light microscopy image of an implant in rat tibia. The bone cortical thickness is small and some areas of bone degeneration are visible. The appearance of the bone is suggestive of osteoporotic conditions. The *red square* represents the region of interest selected for the histomorphometrical measurements. The new bone formation (NB%) was calculated as the area filled with new bone over the area within the red rectangle, excluded the part occupied by the implant (in *red*)

To observe the effects of magnesium in the tissues surrounding the implants, we selected a region of interest (ROI) on each histological slide (Fig. 1) and the amount of new bone formed within that ROI was calculated as percentage (NB%) over the total area of the ROI, excluded the implant, with the software NiS Elements BR 4.30 (Nikon Instruments, Japan).

### Gene expression analysis by means of superarrays

The first step for the gene expression analysis was the purification of ribonucleic acid (RNA) from the cells attached to the implant surfaces. After thawing on ice, all samples were treated with 1× PBS prior to extraction to dissolve salt crystals formed around the implants.

ß-Mercaptoethanol RLT buffer was used to release the cells from the surface of the implants. Total RNA was recovered by spin columns using the RNeasy Micro kit Cat #74004 (QIAGEN, Hilden, Germany), according to manufacturer’s instructions. During extraction, all samples were DNase treated with RNase free DNase Set #79254 (QIAGEN, Hilden, Germany) to reduce gDNA contamination.

The RNA from the samples was reverse transcribed in 20-μl reactions using RT2 First Strand Kit #330401 (SABiosciences, QIAGEN, Hilden, Germany) to generate cDNA. A total of 20 μl of cDNA was generated from each sample.

The cDNA samples were stored in −20 °C until the quantitative polymerase chain reaction (qPCR) was performed.

For the qPCR, RT^2^ Profiler PCR arrays (SABiosciences, QIAGEN, Hilden, Germany), in the format of 96-well plates, were used. The catalogued arrays for Rat Osteogenesis (PARN-026Z) and Rat Osteoporosis (PARN-170Z) were selected for this study. The arrays allow profiling 84 genes of interest at the same time, related to a specific pathway, and they include 5 suggested genes to use as reference and 7 controls (Supplement material Tables 1 and 2).

The qPCR reaction mixture consisted of 1350 μl of 2× RT^2^ SYBR Green Mastermix (SABiosciences, Qiagen, Hilden, Germany), 102 μl of cDNA template, previously diluted with 91 μl of nuclease-free H_2_O and 1248 μl of nuclease-free H_2_O. A total of 25 μl of mixture was added to each well, which contained lyophilized primer assays.

The thermocycling conditions consisted of 10 min at 95 °C and then 40 cycles of 15 s at 95 °C and 1 min at 60 °C of a CFX Connect 96™ thermocycler (Bio-Rad, Hercules, CA, USA), which collected also the fluorescence intensity data after each cycle. Dissociation curves were performed for each well with the following melting curve program: 65 to 95 °C with 0.5 °C step increase for 5 s each step.

The data analysis was performed using the CFX Manager Software 3.0 (Bio-Rad, Hercules, CA, USA) with the normalized relative quantification method. The normalized expression of genes in the test group was calculated relative to the expression in the control group.

### Statistical analysis

Statistical tests used to analyse the influence of the magnesium doping on the bone healing were the non-parametric Wilcoxon signed rank test for the histomorphometrical data (SPSS 22.0 software) and *t* test for the gene expression data (CFX Manager 3.0 Software, Bio-Rad, Hercules, CA, USA). Statistical significance level was set at *p* < 0.05.

## Results

### Surface analysis

**Fig. 2:**
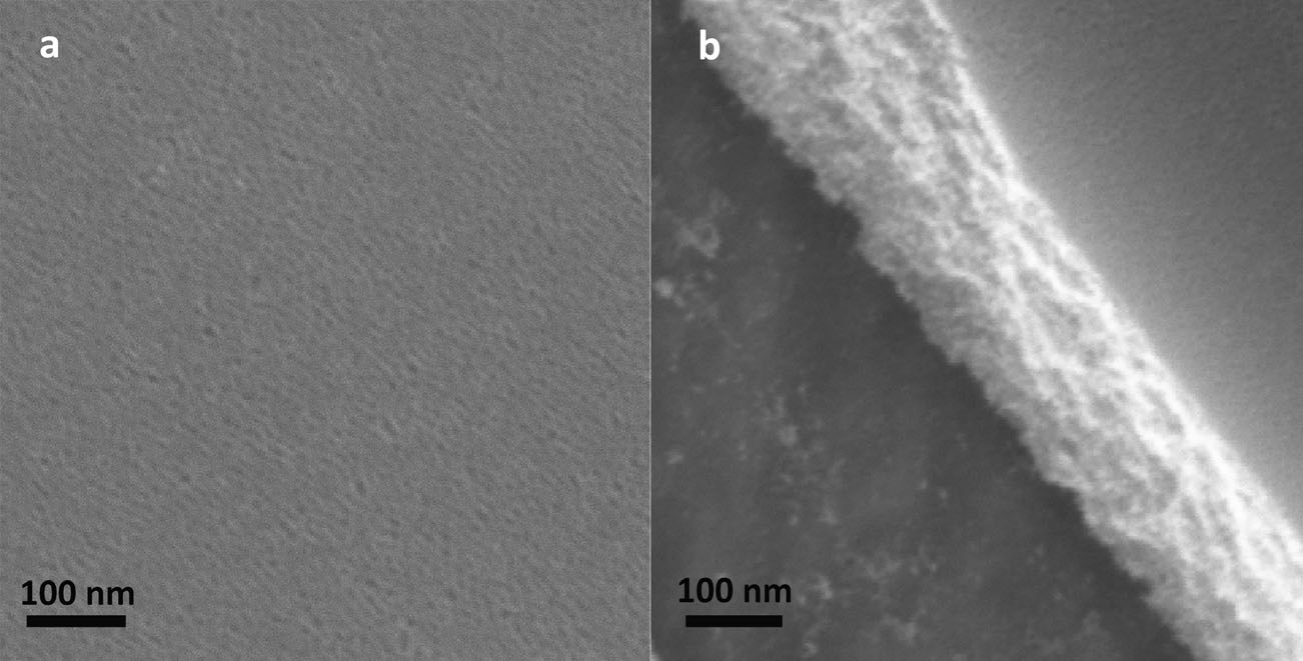
SEM micrographs of the titania mesoporous films on the implant surfaces. **a** The thin mesoporous film shows a high degree of porosity. **b** An area in the mesoporous film has been scratched to visualize the thickness of the coating

A representative image of the thin mesoporous TiO_2_ surface is presented in Fig. 2a. It shows the high porosity level achieved with our treatment, with homogenously distributed pores, facing out from the surface. The coating thickness was assessed in an area with a scratch and was approximately 200 nm (Fig. 2b). No differences were observed between the coatings with or without magnesium ions.

The successful loading of magnesium ions in the mesoporous layers was confirmed by EDX and by ICP-SFMS. The native mesoporous surfaces were composed of oxygen (43.10%), titanium (55.14%) and carbon (5.39%), as detected with EDX. On the mesoporous surfaces loaded with Mg, 0.81% of Mg was detected with the EDX, and the rest was oxygen (47.90%), titanium (45.09%) and carbon (6.18%). The amount of carbon is within the normal level of non-carbon-containing specimens.

The amount of Mg ions in coated implants, measured with ICP-SFMS, was on average 13.09 μg. No Mg was detected in the control implants.

### Histology

The successful induction of the osteoporotic conditions in the ovariectomized rats was confirmed on light microscopy images of the histological sections of the tibiae and femurs. The aspect of the bone in the femoral heads of the rats was trabecular, with big medullar spaces and reduced area occupied by bone, while in the tibia, the cortical region was thin and showed signs of degeneration and the bone density appeared to be considerably low (Fig. 1).

**Fig. 3:**
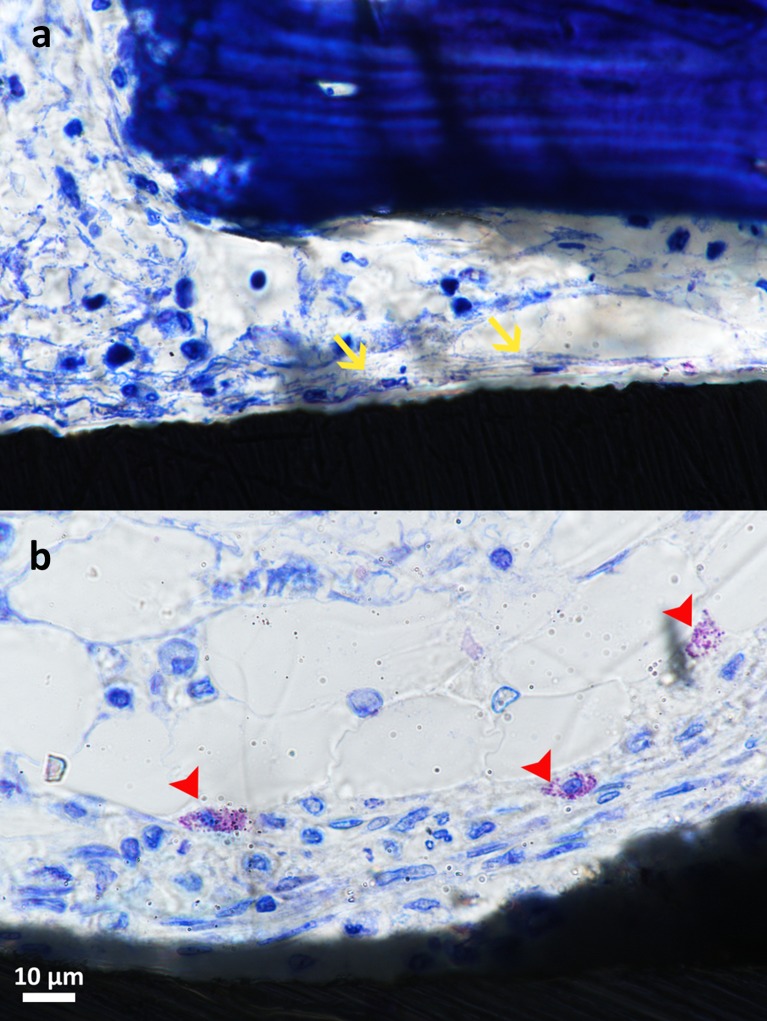
Light microscopy images of histological slides of a magnesium-loaded (**a**) and a control (**b**) specimen 1 day after implantation. A loose network of fibres surrounds the implant surfaces and adheres to them. Extravasated blood cells are recognizable in the blood clot. Cells close to the implant surfaces have a flattened shape and a fibroblast-like aspect (*yellow arrows*). Cells with fine cytoplasm granules suggestive of mast cells are visible in the proximity of the implant (*red arrowheads*). Magnification ×1000. Toluidine blue staining

The images at day 1 of healing revealed that the implant surfaces were surrounded with a loose network of thin fibres, slightly stained, adherent to the implant surfaces. Cells extravasated from blood vessels were found (red blood cells, platelets and leucocytes) within the plot of fibres. Occasionally, osseous fragments, remnants of the drilling process, were found around the implants. The cells close to the implant surfaces had a flattened aspect (Fig. 3a). Cells with fine cytoplasm granules suggestive of mast cells were clearly localized in the proximity of the implants (Fig. 3b). The area of the thin cortical region engaged with the screws showed cracks and bone debris, as a consequence of the drilling process.

No substantial difference was noticed between the test and control implants.

**Fig. 4:**
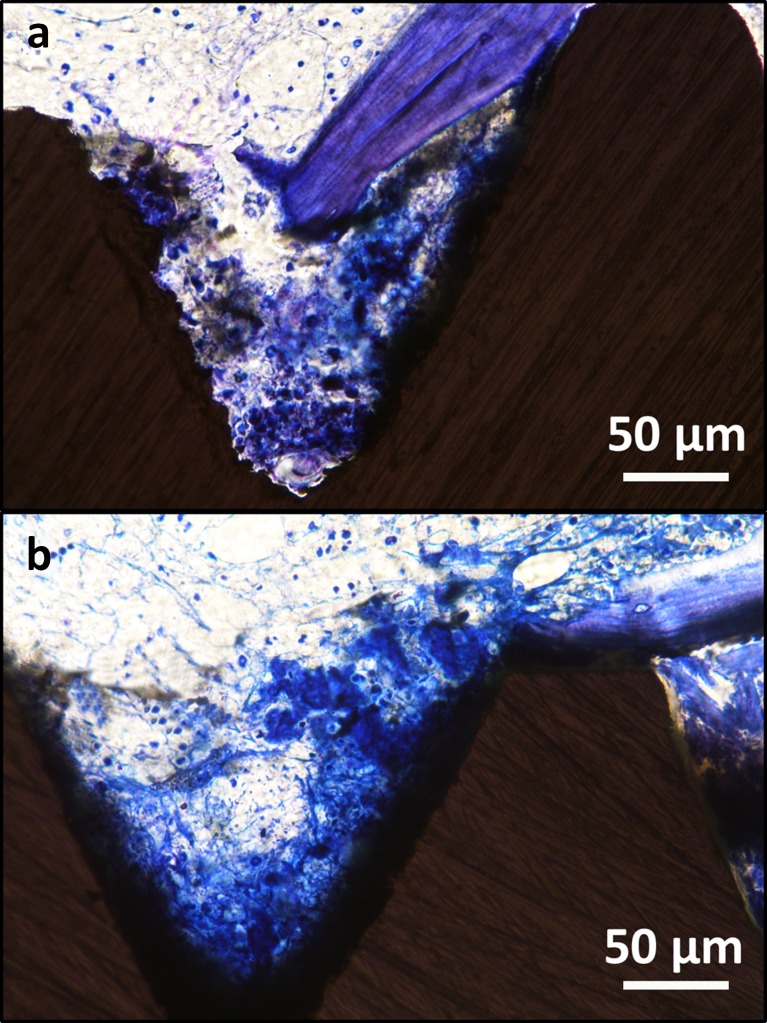
Light microscope images of test (**a**) and control (**b**) specimens after 2 days of healing in rat bone. The blood clot appears more organized and more intensely stained than at day 1. A densely organized fibrin network, containing a great number of cells, adheres to the implants surfaces, especially in correspondence of the implant thread valleys. Magnification ×400. Toluidine blue staining

At day 2, the blood clot appeared thickened and adherent to the implant surfaces, filling the space between the osteotomy and the implant surfaces. The fibrin matrix was thickened on the surfaces of the implants and in the bottom of the threads. The aspect was suggestive of organized blood clot (Fig. 4).

**Fig. 5:**
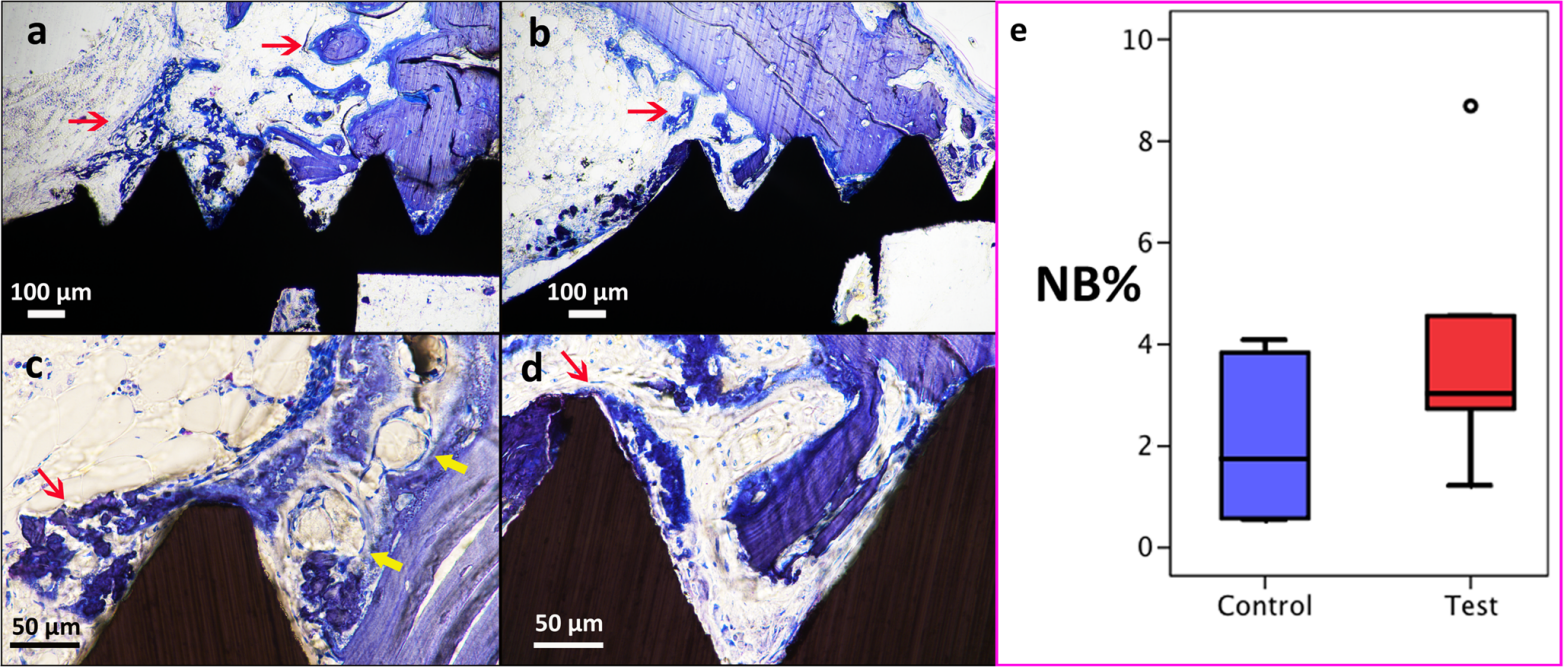
Light microscope images of magnesium-loaded (**a** and **c**) and native (**b** and **d**) mesoporous titania coatings of titanium implants after 7 days of healing. The blood clot is not visible anymore and it has been substituted by a granulation tissue where vascular structures are forming (*yellow arrows*). Woven bone is forming from the surfaces of the mother bone, but also on the surfaces of the implant (*red arrows*). Areas of osteoid formation are observable. Higher amount of new bone is found around the Mg-loaded surfaces compared to the controls. Images **a** and **b** ×100 magnification. Images **c** and **d**, ×400 magnification. Toluidine blue staining. Image **e** displays the box plot (**e**) of the histomorphometrical results of new bone area (NB%) that filled the region of interest. The *central line* of the box plot displays the median of the groups while the *degree sign* displays an outlier of the samples. The difference between the group is statistically significant (*p* = 0.028)

On the specimens at 7 days, it was observed that the blood clot and the granulation tissue were substituted by connective tissue stroma with areas of clearly visible osteoid formation. At this healing time, woven bone was already visible both on the edges of the old bone and bone fragments and at the implant surfaces (Fig. 5). The histomorphometrical analysis revealed that the bone formation was about two times more pronounced around the Mg-releasing implants than around the implants without magnesium (NB% 3.9 vs. 2.1% within the ROI respectively, *p* = 0.028).

### Gene expression

The effect of magnesium release on the expression of genes involved in the pathways of osteogenesis and osteoporosis was investigated by means of qPCR for the cells adherent to the implant surfaces.

The stability of the expression of the five reference genes (ACTB, B2M, HPRT1, LDHA, RPLP1) was checked through an algorithm integrated in the CFX Manager software, and ACTB was excluded as its expression varied too much around samples. The other four genes were suitable (coefficient of variance below 0.5 and M value below 1) and were used for the normalization of the input RNA quantity in each sample.

The internal positive controls indicated no amplification interferences. No amplification curves were detected in the genomic DNA control (RGDC). The values of reverse transcription controls were within the limits suggested by the manufacturer, indicating that the reverse transcription was successful. The melting curves data revealed that the reaction was specific and no primer-dimers were detected.

The amplification was unsuccessful for the gene IHH, meaning that not enough copies of mRNA were originally present in the samples.

The following osteogenesis-related genes were upregulated (fold-change in expression higher than 3) for the magnesium-releasing samples: BMP6, COL14A1, COL2A1, CSF3, VCAM1 and VEGFA; however, only BMP6 reached a level of statistical significance (*R* = 3.8; *p* = 0.027).

**Fig. 6:**
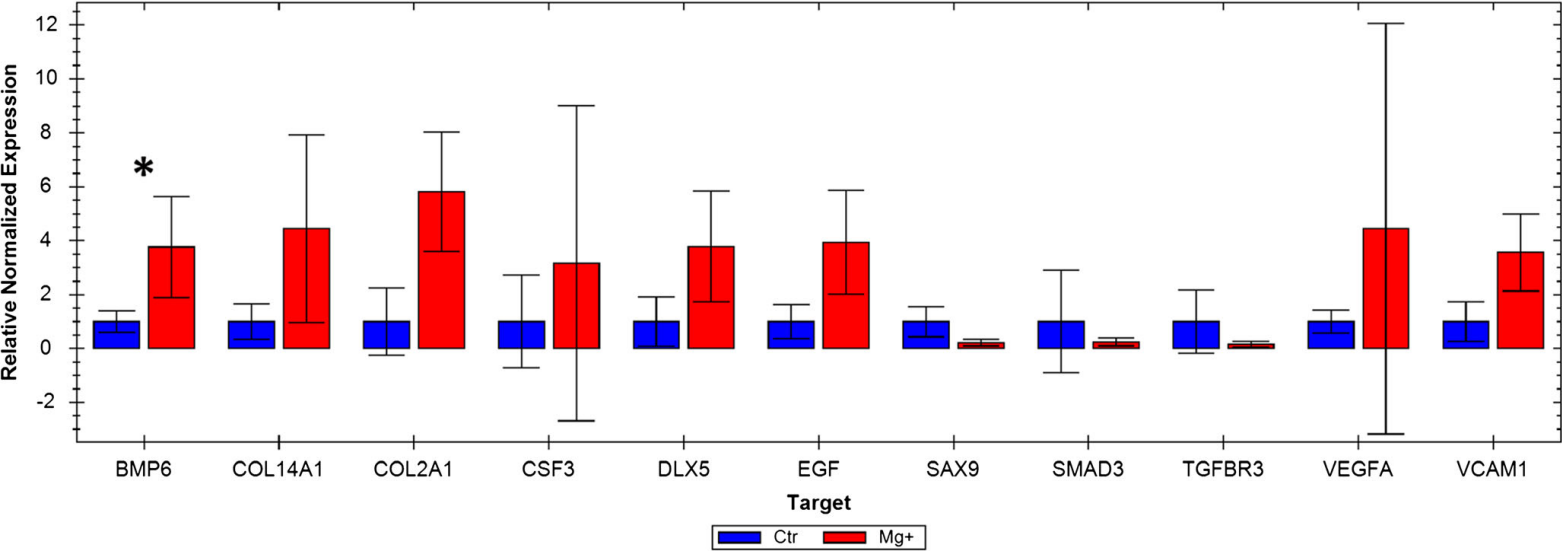
Bar chart of the relative normalized expression of genes involved in the osteogenesis and osteoporosis pathways. Only genes that presented a difference in expression of threefolds or more between the test and the control specimens are displayed. The mRNA quantity for each gene was normalized against four reference genes (B2M, HPRT1, LDHA, RPLP1). * = *p* < 0.05

The downregulated genes were SOX9, SMAD3 and TGFBR3, but none of these genes’ downregulation was significant (Fig. 6).

## Discussion

In previous investigations, the effects of the local administration of Mg ions from mesoporous coatings on the bone anchorage of titanium implants were explored and positive outcomes in healthy bone were noted [22, 23]. Magnesium-doped titanium implants required higher reverse torque strength in order for them to be removed compared to non-doped controls. This biomechanical performance was correlated with higher amount of bone ingrowth within the threads of the magnesium specimens [23]. In addition, the presence of magnesium ions stimulated an osteogenic environment in the peri-implant sites, with upregulation of osteoblastic markers such as osteocalcin, runt-related transcription factor 2 (RUNX2) and insulin growth factor 1 (IGF1) [23].

Based on the outcomes from the previous studies, we tested the hypothesis that sustained release of magnesium ions from mesoporous coatings can be beneficial for the early osseointegration even in compromized situations such as the osteoporosis.

Osteoporotic-like conditions were successfully induced in female rats by means of ovariectomy (OVX) and low-calcium diet for 90 days. Considering the reports that attest the predictability of this protocol in inducing osteoporotic/osteopenic conditions in the rats [32], it was decided to not include sham OVX control animals nor standard diet fed animals for animal use reduction reasons, accordingly with European standards in animal care. As observed on the histological slides, the result of the oestrogen deficiency and of the reduced mineral intake was that the animal showed low bone volume, with thin cortical bone in the tibia and scarce trabeculae both in the tibia and in the femur.

After the insertion of titanium mini-screws coated with mesoporous titania films either with (test) or without (control) Mg ions, we observed a significant increase in woven bone formation during the first week of healing for the Mg-releasing implants compared to the control implants. In addition, the pathway-focused gene expression analysis showed a significant upregulation of BMP6 in the test specimens. Other major genes described as having a role in osteogenesis exhibited major changes in their expression, but the size of our experimental groups did not allow us to demonstrate a statistical significance for these. The statistical sample size has been chosen to characterize the effect of Mg treatment on bone histology, based on our group former experiences with Mg-loaded implants in healthy bones and expecting a comparable effect. In addition, we performed power analysis and we found that a number of 6 samples per group was a sufficient sample size to identify statistical differences in the expression of genes with a large effect size, with a power of 90%.

These results are in accordance with previous findings that the incorporation of approximately 10% of magnesium in hydroxyapatite coatings of titanium screws was found to improve the biological fixation of implants in osteoporotic bone at 12 weeks of healing [33]. However, the molecular pathways involved in the specific cell responses elicited by the magnesium release had not been previously explored in osteoporotic conditions.

The results of the current study suggest that the effect of magnesium could be mediated by BMP6 upregulation. BMP6 is part of the bone morphogenetic family. Similar to other BMPs, BMP6 plays an important role in the induction of osteogenesis. Of interest is that the BMP6 has a functional relationship with 17β-estradiol (E_2_), the female gonadal steroid responsible for osteoporosis in postmenopausal women. The expression of BMP6 in foetal osteoblast cell lines is specifically stimulated by the administration of E_2_, while the other BMPs were less affected [34]. As mentioned above, oestrogen deficiency favours osteoporosis through a diminished control over osteoclasts formation and activity that results in bone loss. But oestrogen deficiency elicits also a reduction in the bone regeneration capacity [35], probably as a consequence of the decreased expression of BMPs, and especially BMP6 [36]. This relationship between BMP6 and oestrogens prompted researchers to test BMP6 as an anabolic agent to promote bone matrix formation in osteoporosis. Interestingly, the systemic administration of BMP6 in ovariectomized rats could restore the bone volume and the bone mechanical characteristics at a level comparable to those of healthy controls [36]. Another study explored the potential of the implantation of stem cells overexpressing BMP6 in vertebral defects of mini pigs and showed that the overexpression of BMP6 induced considerable bone regeneration, while only minor new bone filling was found in the controls [37]. However, some problems exist with regards to the local and systemic application of BMPs due to their high costs and potential side effects of the administration of recombinant growth factors [35].

Thus, the finding that Mg can significantly increase the endogenous BMP6 expression in the vicinity of the implant suggests that Mg doping may be a relative affordable and safe alternative to stimulate an anabolic response around implants placed in osteoporotic sites.

Another reason that may explain the improved performance of the implant with incorporated Mg is that the presence of Mg^2+^ in cell culture media has been reported to promote the direct adhesion of cell pseudopodia to substrates, probably via an integrin-mediated mechanism [38]. Mg ions are more effective than any other divalent cations (calcium, strontium and barium) in inducing cell attachment and spreading to different substrates [39]. This was confirmed in a study in which polished titanium plates were hydrothermally treated with MgCl_2_ solution, and gingival epithelial cells and fibroblastic cells were cultured on them to test for cellular adhesion [40]. In the study, it was shown that the Mg groups displayed a much stronger attachment of fibroblastic cells, but not of epithelial cells, to the titanium substrate compared to untreated and to CaCl_2_-treated titanium plates [40]. The fibroblasts showed higher development of actin filament, more lamellopodia and higher signal of cytoplasmic vinculin, which is a sign of attachment [40]. Similarly in the current study, we could observe flattened fibroblastic-like cells on the surfaces of the Mg-treated implants as soon as day 1 and it was possible to notice a slight increase of attachment for the Mg-loaded surfaces, although the differences were not statistically significant. Especially in those cases with reduced bone volume and consequent risk of implant micromotion, the promotion of cell adhesion and spreading may be beneficial for rapid osseointegration, decreasing the risk of implant loosening.

One further benefit of Mg on bone health is the stimulation of vascularization [27]. It has been suggested that magnesium deficiency can cause decrease in blood vessels formation and in their volumes and thus in blood supply, which can be an indirect cause of osteoporosis development [27]. On the contrary, Mg supplementation helps endothelial cell proliferation and function [41], which may well contribute to rapid osseointegration. In line with that hypothesis, we observed a faster organization of new blood vessels around the test implants and also a marked upregulation of VEGFA, a growth factor for vascular endothelial cells, despite the difference was not significant.

Furthermore, it has been discovered that VEGF produced by osteoblasts is a potentially crucial factor for the differentiation of MSCs toward bone forming cells rather and to adipocytes [42]. The mechanism leading to this effect is intracrine, meaning that the VEGF released by the osteoblasts produces an effect on osteoblasts themselves. Knock-out mice for this factor did show osteoporotic bone and higher amount of fat cells in the bone marrow, as compared to a normal individual [42]. Therefore, impairment in VEGF production could be another pathway that leads to the onset of the osteoporotic conditions in patients. In the current study, the release of Mg ions from the implant surfaces stimulated the expression of this gene too and that could be correlated to a higher amount of new bone formation compared to controls without Mg.

We observed histologically a similar progression of healing for the test and control groups. One day after insertion of the implants, hematoma was the predominant aspect in the tissues around the implants. Cells extravasated from the interrupted blood vessels, especially red blood cells, platelets and leucocytes, were found in the areas around the implants and within the matrix of fibrin. As well, leucocyte residents in the bone and bone marrow, such as mast cells and polymorphonuclear leucocytes, were found in the proximity of the implants. All these cells and fibrin filaments are known to contribute to the formation of the blood clot and successively to the activation and the recruitment of stromal and endothelial cells for the healing of the defect and the formation of an interface with the implant surfaces [43]. After 7 days, the initially formed hematoma and blood clot were substituted by bone and blood vessels forming cells and newly formed bone was visible around the implant surfaces. The amount of new bone formation was significantly higher for the magnesium-releasing surfaces compared to the native mesoporous titania substrates, suggesting that the release of magnesium ions stimulates a faster bone formation in the first days of healing.

We chose to investigate the cellular and molecular effects of magnesium in the initial phases of the establishment of bone fixation because we hypothesized that the influence of magnesium release would have been the greatest shortly after implant insertion. A recent work from Cecchinato and colleagues, in which mesoporous titania thin films were coated on a quartz crystal and adsorption and release profile of Mg ions were studied using quartz crystal microbalance with dissipation (QCM-D), showed that the mobilization of Mg ions from the mesoporous carriers is initially rapid and then it is followed by a sustained release [44]. It was therefore speculated that this burst release would interact with the surrounding environment immediately after implant insertion. The selection of these short time points for evaluations is a potential limitation of the present investigation, as it does not provide information on the long-term performance of osseointegration of the studied implants. However, the rat is known to have a much faster bone turnover than humans and, therefore, 7 days in rats represent a longer healing time in humans. In addition, evidence from clinical studies showed that osteoporosis does not decrease the long-performance of implants, when osseointegration is already established, but can constitute a risk of failure during the early phases of implant healing, due to the higher instability of implants placed in low density bone and, thus, the increased possibility of micromotions [9]. Therefore, a surface modification which increases the bone formation and the bone mineralization in the first weeks of healing might be of interest to reduce the risk of early implant failure, especially for those patients with other concomitant conditions, as smoking or bruxism. Furthermore, the positive effects of magnesium doping on the amount and strength of osseointegration were observable even at longer healing times in a rabbit healthy bone [22, 23].

Another potential limitation of the present study is the substantial difference in bone metabolism, bone remodelling and bone anatomy of the rat compared to humans, as for example rat bone lacks a well-developed Haversian canal system, which is present in human bone [32]. However, the rat is still considered the elective animal model in orthopaedic research for the study of osteopenic and osteoporotic bone, thanks to the extensive knowledge that exists on the rat skeleton and the possibility to mimic the human postmenopausal osteoporotic conditions in rats with very good predictability [32]. For this reason, the US Food and Drug Administration recommends that the pre-clinical evaluation of agents for the prevention or treatments of osteoporosis always includes tests in ovariectomized rats.

In the current study, it was decided to employ a tibia and femur model to test implant osseointegration. A disadvantage of the selection of long bone models to study oral and maxillofacial implants is that the implants could not be prosthetically loaded during the course of the study. It can be hypothesized that the implants did not undergo the same biomechanical stimulation as in the jaws, making it difficult to generalize the results for the clinical situations. However, it has been suggested that the morphology of the proximal tibia resembles that of the anterior mandible, with a thick cortical bone encapsulating bone marrow, and the distal femoral epiphysis resembles the less dense trabecular bone of the posterior maxilla [45]. These regions are also easier to access and larger implants can be installed there than in rat jaws. Therefore, tibia and femur models are considered an adequate approximation of the intra-oral anatomical locations and they are extensively employed to investigate the tissue compatibility and bioactivity of oral implants [45]. In addition, the current study aimed to give and insight through gene expression analysis to the molecular processes of bone healing that occur in the bone in proximity of the implant surfaces in correlation with Mg release and it can be speculated that similar pathways are activated in distinct anatomical locations.

The potential benefits of Mg ions release in inducing the early formation of a bone-to-implant interface in bone sites with osteoporosis make this surface treatment an interesting candidate for continued studies. The further development and implementation of Mg-releasing surfaces, tailored for implantation in compromized situations, may lead to improvement of implant fixation in those patients with osteoporosis.

## Conclusions

We evaluated the influence of a local release of Mg ions from mesoporous titania films on the early phases of implant fixation in an osteoporotic animal model. The results showed that, subsequently after implant placement, the coagulum formed around the implants. Woven bone formation was already visible after 7 days of implant installation and the new bone volume around the screws was significantly higher for the specimens with magnesium.

The gene expression profiling of markers involved in the pathways of osteogenesis and osteoporosis revealed a significant upregulation of BMP6, a marker specifically associated with oestrogen and with a strong anabolic activity in osteoporotic bone, around the magnesium-doped surfaces.

Despite some care should be applied before generalizing these results to the clinical situation, considering the selection of a rodent model compared to human, the current data indicate that the addition of magnesium in mesoporous implant coatings may enhance the initial stimulation of osseointegration in patients with osteoporosis.

## Electronic supplementary material

Table S1 (DOCX 18 kb).

Table S2 (DOCX 19 kb).
