# Correction to: The effect of magnesium on early osseointegration in osteoporotic bone: a histological and gene expression investigation

**DOI:** 10.1007/s00198-018-4497-6

**Published:** 2018-07-03

**Authors:** S. Galli, M. Stocchero, M. Andersson, J. Karlsson, W. He, T. Lilin, A. Wennerberg, R. Jimbo

**Affiliations:** 1 Department of Prosthodontics, Faculty of Odontology, Malmö University, Malmö, Sweden; 2 Department of Oral and Maxillofacial Surgery and Oral Medicine, Faculty of Odontology, Malmö University, Malmö, Sweden; 3 Department of Chemistry and Chemical Engineering, Applied Chemistry, Chalmers University of Technology, Gothenburg, Sweden; 4 Center for Biomedical Research, ECole Nationale Vétérinaire d’Alfort, Maisons Alfort, France

**Correction to: Osteoporos Int**

10.1007/s00198-017-4004-5

This article was originally published under a CC BY-NC-ND 4.0 license, but has now been made available under a CC BY 4.0 license. The PDF and HTML versions of the paper have been modified accordingly.

