# Supplementary material for: The effect of magnesium on early osseointegration in osteoporotic bone: a histological and gene expression investigation
Source: Osteoporos Int. 2017 Mar 27;28(7):2195–205. doi: 10.1007/s00198-017-4004-5 (PMC5486930; doi:10.1007/s00198-017-4004-5)
Supplement: Supplementary file 1 — (DOCX 18 kb). [file 198_2017_4004_MOESM1_ESM.docx]

Table 1: List of genes and controls included in the RT^2^ Profiler PCR array for Rat Osteogenesis, Qiagen.

| **Gene description** | **Symbol** | **Refseq according to** | **Gname** |
| --- | --- | --- | --- |
| Activin A receptor, type I | ACVR1 | NM_024486 | - |
| Alpha-2-HS-glycoprotein | AHSG | NM_012898 | AA2-066/PP63 |
| Alkaline phosphatase, liver/bone/kidney | ALPL | NM_013059 | AKP2/PHOA |
| Annexin A5 | ANXA5 | NM_013132 | ANX5/LC5 |
| Bone gamma-carboxyglutamate (gla) protein | BGLAP | NM_013414 | BGLAP2/BGP/BGPR/BGPRA |
| Biglycan | BGN | NM_017087 | BSPG1 |
| Bone morphogenetic protein 1 | BMP1 | NM_031323 | - |
| Bone morphogenetic protein 2 | BMP2 | NM_017178 | - |
| Bone morphogenetic protein 3 | BMP3 | NM_017105 | PBMP3 |
| Bone morphogenetic protein 4 | BMP4 | NM_012827 | BOMPR4A |
| Bone morphogenetic protein 5 | BMP5 | NM_001108168 | - |
| Bone morphogenetic protein 6 | BMP6 | NM_013107 | VGR |
| Bone morphogenetic protein 7 | BMP7 | NM_001191856 | BMP-7 |
| Bone morphogenetic protein receptor, type IA | BMPR1A | NM_030849 | - |
| Bone morphogenetic protein receptor, type IB | BMPR1B | NM_001024259 | CFK-43A |
| Bone morphogenetic protein receptor, type II (serine/threonine kinase) | BMPR2 | NM_080407 | BMPR-II |
| CD36 molecule (thrombospondin receptor) | CD36 | NM_031561 | FAT |
| Cadherin 11 | CDH11 | NM_053392 | - |
| Chordin | CHRD | NM_057134 | - |
| Collagen, type X, alpha 1 | COL10A1 | XM_001053056 | - |
| Collagen, type XIV, alpha 1 | COL14A1 | NM_001130548 | - |
| Collagen, type I, alpha 1 | COL1A1 | NM_053304 | COLIA1 |
| Collagen, type I, alpha 2 | COL1A2 | NM_053356 | - |
| Collagen, type II, alpha 1 | COL2A1 | NM_012929 | CG2A1A/COLLII |
| Collagen, type III, alpha 1 | COL3A1 | NM_032085 | - |
| Collagen, type IV, alpha 1 | COL4A1 | NM_001135009 | - |
| Collagen, type V, alpha 1 | COL5A1 | NM_134452 | - |
| Collagen, type VI, alpha 1 | COL6A1 | XM_215375 | RGD1565398 |
| Cartilage oligomeric matrix protein | COMP | NM_012834 | - |
| Colony stimulating factor 1 (macrophage) | CSF1 | NM_023981 | - |
| Colony stimulating factor 2 (granulocyte-macrophage) | CSF2 | NM_053852 | GM-CSF/GMCSF |
| Colony stimulating factor 3 (granulocyte) | CSF3 | NM_017104 | GCSF |
| Cathepsin K | CTSK | NM_031560 | - |
| Distal-less homeobox 5 | DLX5 | NM_012943 | RDLX |
| Epidermal growth factor | EGF | NM_012842 | - |
| Fibroblast growth factor 1 | FGF1 | NM_012846 | FGF-1/HBGF-1/HBGF1 |
| Fibroblast growth factor 2 | FGF2 | NM_019305 | FGF-2/BFGF |
| Fibroblast growth factor receptor 1 | FGFR1 | NM_024146 | - |
| Fibroblast growth factor receptor 2 | FGFR2 | NM_001109892 | - |
| Fms-related tyrosine kinase 1 | FLT1 | NM_019306 | VEGFR-1 |
| Fibronectin 1 | FN1 | NM_019143 | FIBNEC/FN-1 |
| Growth differentiation factor 10 | GDF10 | NM_024375 | - |
| GLI family zinc finger 1 | GLI1 | NM_001191910 | GLI |
| Intercellular adhesion molecule 1 | ICAM1 | NM_012967 | CD54/ICAM |
| Insulin-like growth factor 1 | IGF1 | NM_178866 | - |
| Insulin-like growth factor 1 receptor | IGF1R | NM_052807 | IGFIRC/JTK13 |
| Indian hedgehog | IHH | NM_053384 | - |
| Integrin, alpha 2 | ITGA2 | XM_345156 | CD49B |
| Integrin, alpha 3 | ITGA3 | NM_001108292 | - |
| Integrin, alpha M | ITGAM | NM_012711 | CD11B |
| Integrin, alpha V | ITGAV | NM_001106549 | CD51 |
| Integrin, beta 1 | ITGB1 | NM_017022 | - |
| Matrix metallopeptidase 10 | MMP10 | NM_133514 | - |
| Matrix metallopeptidase 2 | MMP2 | NM_031054 | - |
| Matrix metallopeptidase 8 | MMP8 | NM_022221 | - |
| Matrix metallopeptidase 9 | MMP9 | NM_031055 | - |
| Nuclear factor of kappa light polypeptide gene enhancer in B-cells 1 | NFKB1 | NM_001276711 | EBP-1/NF-KB |
| Noggin | NOG | NM_012990 | - |
| Platelet-derived growth factor alpha polypeptide | PDGFA | NM_012801 | PDGFACP |
| Phosphate regulating endopeptidase homolog, X-linked | PHEX | NM_013004 | PEX |
| Runt-related transcription factor 2 | RUNX2 | NM_053470 | CBFA1/OSF-2 |
| Serine (or cysteine) peptidase inhibitor, clade H, member 1 | SERPINH1 | NM_017173 | CBP2/SERPINH2 |
| SMAD family member 1 | SMAD1 | NM_013130 | MADH1 |
| SMAD family member 2 | SMAD2 | NM_019191 | MADH2 |
| SMAD family member 3 | SMAD3 | NM_013095 | MADH3/SMAD 3/MAD3 |
| SMAD family member 4 | SMAD4 | NM_019275 | MADH4 |
| SMAD family member 5 | SMAD5 | NM_021692 | MADH5 |
| Sclerosteosis | SOST | NM_030584 | - |
| SRY-box containing gene 9 | SOX9 | XM_001081628 | - |
| Sp7 transcription factor | SP7 | NM_181374 | OSX |
| Secreted phosphoprotein 1 | SPP1 | NM_012881 | OSP |
| Transforming growth factor, beta 1 | TGFB1 | NM_021578 | TGFB |
| Transforming growth factor, beta 2 | TGFB2 | NM_031131 | TGF-B2 |
| Transforming growth factor, beta 3 | TGFB3 | NM_013174 | TGF-B3 |
| Transforming growth factor, beta receptor 1 | TGFBR1 | NM_012775 | ALK5/SKR4/TGFR-1/TBETAR-I |
| Transforming growth factor, beta receptor II | TGFBR2 | NM_031132 | TGF-BETA 2/TGFBR2T |
| Transforming growth factor, beta receptor III | TGFBR3 | NM_017256 | BETAGLYCAN |
| Tumor necrosis factor (TNF superfamily, member 2) | TNF | NM_012675 | RATTNF/TNF-ALPHA/TNFA |
| Tumor necrosis factor (ligand) superfamily, member 11 | TNFSF11 | NM_057149 | RANKL |
| Twist homolog 1 (Drosophila) | TWIST1 | NM_053530 | TWIST |
| Vascular cell adhesion molecule 1 | VCAM1 | NM_012889 | VCAM1B |
| Vitamin D (1,25- dihydroxyvitamin D3) receptor | VDR | NM_017058 | NR1I1 |
| Vascular endothelial growth factor A | VEGFA | NM_031836 | VEGF-A/VEGF164/VPF/VEGF |
| Vascular endothelial growth factor B | VEGFB | NM_053549 | - |
| Actin, beta | ACTB | NM_031144 | ACTX |
| Beta-2 microglobulin | B2M | NM_012512 | - |
| Hypoxanthine phosphoribosyltransferase 1 | HPRT1 | NM_012583 | HGPRTASE/HPRT |
| Lactate dehydrogenase A | LDHA | NM_017025 | LDH1 |
| Ribosomal protein, large, P1 | RPLP1 | NM_001007604 | - |
| Rat Genomic DNA Contamination | RGDC | U26919 | RGDC |
| Reverse Transcription Control | RTC | SA_00104 | RTC |
| Reverse Transcription Control | RTC | SA_00104 | RTC |
| Reverse Transcription Control | RTC | SA_00104 | RTC |
| Positive PCR Control | PPC | SA_00103 | PPC |
| Positive PCR Control | PPC | SA_00103 | PPC |
| Positive PCR Control | PPC | SA_00103 | PPC |
