# Supplementary material for: The effect of magnesium on early osseointegration in osteoporotic bone: a histological and gene expression investigation
Source: Osteoporos Int. 2017 Mar 27;28(7):2195–205. doi: 10.1007/s00198-017-4004-5 (PMC5486930; doi:10.1007/s00198-017-4004-5)
Supplement: Supplementary file 2 — (DOCX 19 kb). [file 198_2017_4004_MOESM2_ESM.docx]

Table 2: List of genes and controls included in the RT^2^ Profiler PCR array for Rat Osteoporosis, Qiagen.

| **Description** | **Symbol** | **Refseq** | **Gname** |
| --- | --- | --- | --- |
| Acid phosphatase 5, tartrate resistant | Acp5 | NM_019144 | TTRRAP/Trap |
| Adenylate cyclase 10 (soluble) | Adcy10 | NM_021684 | Sac |
| Arachidonate 12-lipoxygenase | Alox12 | NM_001105798 | 12-LO/12S-LOX |
| Arachidonate 15-lipoxygenase | Alox15 | NM_031010 | 12-LOX/15-LOX/Alox12/Alox12l |
| Arachidonate 5-lipoxygenase | Alox5 | NM_012822 | LOX5A |
| Alkaline phosphatase, liver/bone/kidney | Alpl | NM_013059 | Akp2/PHOA |
| Androgen receptor | Ar | NM_012502 | Andr/Tfm |
| Bone gamma-carboxyglutamate (gla) protein | Bglap | NM_013414 | Bglap2/Bgp/Bgpr/Bgpra |
| Bone morphogenetic protein 2 | Bmp2 | NM_017178 | - |
| Bone morphogenetic protein 7 | Bmp7 | NM_001191856 | BMP-7 |
| Calcitonin-related polypeptide alpha | Calca | NM_017338 | CAL6/CGRP/Cal1/Calc/RATCAL6/calcitonin |
| Calcitonin receptor | Calcr | NM_053816 | - |
| Carbonic anhydrase II | Car2 | NM_019291 | Ca2 |
| Calcium-sensing receptor | Casr | NM_016996 | PCaR1 |
| CD40 molecule, TNF receptor superfamily member 5 | Cd40 | NM_134360 | Tnfrsf5 |
| Chloride channel 7 | Clcn7 | NM_031568 | ClC-7 |
| Cannabinoid receptor 2 (macrophage) | Cnr2 | NM_020543 | CB-2/CB2/CB2C/CNR2C |
| Collagen, type I, alpha 1 | Col1a1 | NM_053304 | COLIA1 |
| Collagen, type I, alpha 2 | Col1a2 | NM_053356 | - |
| Catechol-O-methyltransferase | Comt | NM_012531 | - |
| Cartilage associated protein | Crtap | NM_001108785 | RGD1565180 |
| Cathepsin K | Ctsk | NM_031560 | - |
| Cytochrome P450, family 17, subfamily a, polypeptide 1 | Cyp17a1 | NM_012753 | Cyp17 |
| Cytochrome P450, family 19, subfamily a, polypeptide 1 | Cyp19a1 | NM_017085 | Aromatase/Cyp19/Cyp19a/p450arom |
| D site of albumin promoter (albumin D-box) binding protein | Dbp | NM_012543 | - |
| Dickkopf homolog 1 (Xenopus laevis) | Dkk1 | NM_001106350 | - |
| Ectonucleotide pyrophosphatase/phosphodiesterase 1 | Enpp1 | NM_053535 | Npps/Pc1 |
| Estrogen receptor 1 | Esr1 | NM_012689 | ER-alpha/Esr/RNESTROR |
| Estrogen receptor 2 (ER beta) | Esr2 | NM_012754 | ER-beta/ERbeta/Erb2 |
| Estrogen related receptor, alpha | Esrra | NM_001008511 | ERRalpha/Errra |
| Fibroblast growth factor receptor 1 | Fgfr1 | NM_024146 | - |
| Fibroblast growth factor receptor 2 | Fgfr2 | NM_001109892 | - |
| Growth hormone releasing hormone | Ghrh | NM_031577 | GHRF |
| Hydroxysteroid 11-beta dehydrogenase 1 | Hsd11b1 | NM_017080 | LRRGT00065 |
| Insulin-like growth factor 1 | Igf1 | NM_178866 | - |
| Insulin-like growth factor binding protein 2 | Igfbp2 | NM_013122 | BRL-BP/IBP-2/IGFBP-2/ILGFBPA |
| Interleukin 15 | Il15 | NM_013129 | - |
| Interleukin 6 | Il6 | NM_012589 | ILg6/Ifnb2 |
| Interleukin 6 receptor | Il6r | NM_017020 | IL6R1/Il6ra |
| Integrin, alpha 1 | Itga1 | NM_030994 | - |
| Integrin, beta 3 | Itgb3 | NM_153720 | - |
| Leptin | Lep | NM_013076 | OB/obese |
| Leucine proline-enriched proteoglycan (leprecan) 1 | P3h1 | NM_053667 | Gros1/Lepre1 |
| Similar to Tumor necrosis factor, alpha-induced protein 3 (Putative DNA binding protein A20) (Zinc finger protein A20) | Tnfaip3 | XM_003748656 | - |
| Low density lipoprotein-related protein 1 (alpha-2-macroglobulin receptor) | Lrp1 | NM_001130490 | - |
| Low density lipoprotein receptor-related protein 5 | Lrp5 | NM_001106321 | - |
| Low density lipoprotein receptor-related protein 6 | Lrp6 | NM_001107892 | - |
| Lymphotoxin alpha (TNF superfamily, member 1) | Lta | NM_080769 | Tnfb |
| Latent transforming growth factor beta binding protein 2 | Ltbp2 | NM_021586 | - |
| Mab-21-like 2 (C. elegans) | Mab21l2 | NM_001109391 | - |
| Matrix metallopeptidase 2 | Mmp2 | NM_031054 | - |
| Myostatin | Mstn | NM_019151 | Gdf8 |
| Methylenetetrahydrofolate reductase (NAD(P)H) | Mthfr | XM_342975 | - |
| Nuclear factor of activated T-cells, cytoplasmic, calcineurin-dependent 1 | Nfatc1 | NM_001244933 | - |
| Noggin | Nog | NM_012990 | - |
| Nitric oxide synthase 3, endothelial cell | Nos3 | NM_021838 | eNos |
| Neuropeptide Y | Npy | NM_012614 | NPY02/RATNPY/RATNPY02 |
| Nuclear receptor subfamily 3, group C, member 1 | Nr3c1 | NM_012576 | GR/Gcr/Grl |
| Purinergic receptor P2X, ligand-gated ion channel, 7 | P2rx7 | NM_019256 | - |
| Procollagen lysine, 2-oxoglutarate 5-dioxygenase 2 | Plod2 | NM_175869 | - |
| Prolactin | Prl | NM_012629 | PRLB/PRLSD1/Prl1a1/Prol/RATPRLSD1/RNPROL |
| Parathyroid hormone | Pth | NM_017044 | PTH-(1-84)/Pth1/Pthr1 |
| Parathyroid hormone 1 receptor | Pth1r | NM_020073 | PTHrel/Pthr/Pthr1 |
| Parathyroid hormone-like hormone | Pthlh | NM_012636 | PLP |
| Runt-related transcription factor 2 | Runx2 | NM_053470 | Cbfa1/OSF-2 |
| Secreted frizzled-related protein 1 | Sfrp1 | NM_001276712 | sFRP-1 |
| Secreted frizzled-related protein 4 | Sfrp4 | NM_053544 | - |
| Sex hormone binding globulin | Shbg | NM_012650 | Abpa |
| Sclerosteosis | Sost | NM_030584 | - |
| Secreted protein, acidic, cysteine-rich (osteonectin) | Sparc | NM_012656 | - |
| Secreted phosphoprotein 1 | Spp1 | NM_012881 | OSP |
| Signal transducer and activator of transcription 1 | Stat1 | NM_032612 | DD6G4-4 |
| Transforming growth factor, beta 1 | Tgfb1 | NM_021578 | Tgfb |
| TIMP metallopeptidase inhibitor 2 | Timp2 | NM_021989 | - |
| Tumor necrosis factor receptor superfamily, member 11a, NFKB activator | Tnfrsf11a | NM_001271235 | RANK/RGD1563614 |
| Tumor necrosis factor receptor superfamily, member 11b | Tnfrsf11b | NM_012870 | Opg |
| Tumor necrosis factor receptor superfamily, member 1b | Tnfrsf1b | NM_130426 | Tnfr2 |
| Tumor necrosis factor (ligand) superfamily, member 11 | Tnfsf11 | NM_057149 | RANKL |
| Thyroid stimulating hormone receptor | Tshr | NM_012888 | TSHRA |
| Twist homolog 1 (Drosophila) | Twist1 | NM_053530 | Twist |
| Vitamin D (1,25- dihydroxyvitamin D3) receptor | Vdr | NM_017058 | Nr1i1 |
| Vascular endothelial growth factor A | Vegfa | NM_031836 | VEGF-A/VEGF164/VPF/Vegf |
| Wingless-type MMTV integration site family, member 10B | Wnt10b | NM_001108111 | - |
| Wingless-type MMTV integration site family, member 3A | Wnt3a | NM_001107005 | - |
| Actin, beta | Actb | NM_031144 | Actx |
| Beta-2 microglobulin | B2m | NM_012512 | - |
| Hypoxanthine phosphoribosyltransferase 1 | Hprt1 | NM_012583 | Hgprtase/Hprt |
| Lactate dehydrogenase A | Ldha | NM_017025 | Ldh1 |
| Ribosomal protein, large, P1 | Rplp1 | NM_001007604 | - |
| Rat Genomic DNA Contamination | RGDC | U26919 | RGDC |
| Reverse Transcription Control | RTC | SA_00104 | RTC |
| Reverse Transcription Control | RTC | SA_00104 | RTC |
| Reverse Transcription Control | RTC | SA_00104 | RTC |
| Positive PCR Control | PPC | SA_00103 | PPC |
| Positive PCR Control | PPC | SA_00103 | PPC |
| Positive PCR Control | PPC | SA_00103 | PPC |
